# Inhibition of STAT3, FAK and Src mediated signaling reduces cancer stem cell load, tumorigenic potential and metastasis in breast cancer

**DOI:** 10.1038/srep10194

**Published:** 2015-05-14

**Authors:** Ravi Thakur, Rachana Trivedi, Namrata Rastogi, Manisha Singh, Durga Prasad Mishra

**Affiliations:** 1 Cell Death Research Laboratory, Endocrinology Division, CSIR-CDRI, Lucknow, INDIA

## Abstract

Cancer stem cells (CSCs) are responsible for aggressive tumor growth, metastasis and therapy resistance. In this study, we evaluated the effects of Shikonin (Shk) on breast cancer and found its anti-CSC potential. Shk treatment decreased the expression of various epithelial to mesenchymal transition (EMT) and CSC associated markers. Kinase profiling array and western blot analysis indicated that Shk inhibits STAT3, FAK and Src activation. Inhibition of these signaling proteins using standard inhibitors revealed that STAT3 inhibition affected CSCs properties more significantly than FAK or Src inhibition. We observed a significant decrease in cell migration upon FAK and Src inhibition and decrease in invasion upon inhibition of STAT3, FAK and Src. Combined inhibition of STAT3 with Src or FAK reduced the mammosphere formation, migration and invasion more significantly than the individual inhibitions. These observations indicated that the anti-breast cancer properties of Shk are due to its potential to inhibit multiple signaling proteins. Shk also reduced the activation and expression of STAT3, FAK and Src *in vivo* and reduced tumorigenicity, growth and metastasis of 4T1 cells. Collectively, this study underscores the translational relevance of using a single inhibitor (Shk) for compromising multiple tumor-associated signaling pathways to check cancer metastasis and stem cell load.

Breast cancer is the most common endocrine cancer and the second leading cause of cancer-related deaths in women. In spite of the diverse therapeutic regimens available for breast cancer treatment, development of chemo-resistance and disease relapse is constantly on the rise. The most common cause of disease relapse and chemo-resistance is attributed to the presence of stem cell like cells (or CSCs) in tumor tissues^1^,^2^. CSCs represent a small population within the tumor mass, capable of inducing independent tumors *in vivo* and are hard to eradicate^2^. Multiple signaling pathways including Receptor Tyrosine Kinase (RTKs), Wnt/β-catenin, TGF-β, STAT3, Integrin/FAK, Notch and Hedgehog signaling pathway helps in maintaining the stem cell programs in normal as well as in cancer cells^3^,^4^,^5^,^6^. These pathways also support the epithelial-mesenchymal transition (EMT) and expression of various drug transporters in cancer cells. Cells undergoing EMT are known to acquire stem cell and chemo-resistant traits^7^. Thus, the induction of EMT programs, drug resistance and stem cell like properties are interlinked^7^. Commonly used anti-cancer drugs eradicate most of the tumor cells, but CSCs due to their robust survival mechanisms remain viable and lead to disease relapse^8^. Studies carried out on patient derived tumor samples and *in vivo* mouse models have demonstrated that the CSCs metastasize very efficiently than non-CSCs^9^,^10^,^11^. Therefore, drugs capable of compromising CSCs proliferation and self-renewal are urgently required as the inhibition of CSC will induce the inhibition of tumor growth, chemo-resistance, metastasis and metastatic colonization in breast cancer.

Shikonin, a natural dietary component is a potent anti-cancer compound^12^,^13^. Previous studies have shown that Shk inhibits the cancer cell growth, migration, invasion and tumorigenic potential^12^. Shk has good bioavailability, less toxicity and favorable pharmacokinetic and pharmacodynamic profiles *in vivo*^12^. In a recent report, it was shown that the prolonged exposure of Shk to cancer cells does not cause chemo-resistance^13^.Other studies have shown that it inhibits the expression of various key inflammatory cytokines and associated signaling pathways^12^,^14^. It decreases the expression of TNFα, IL12, IL6, IL1β, IL2, IFNγ, inhibits ERK1/2 and JNK signaling and reduces the expression of NFκB and STAT3 transcription factors^14^,^15^. It inhibits proteasome and also modulates the cancer cell metabolism by inhibiting tumor specific pyurvate kinase-M2^14^,^15^,^16^. Skh causes cell cycle arrest and induces necroptosis in various cancer types^14^. Shk also inhibits the expression of MMP9, integrin β1 and decreases invasive potential of cancer cells^14^,^17^. Collectively, Shk modulates various signaling pathways and elicits anti-cancer responses in a variety of cancer types.

In breast cancer, Shk has been reported to induce the cell death and inhibit cell migration, but the mechanisms responsible for its effect are not well studied^18^,^19^. Signaling pathways modulated by Shk in cancerous and non-cancerous models have previously been shown important for breast cancer growth, metastasis and tumorigenicity^20^. Therefore in the current study, we investigated the effect of Shk on various hallmark associated properties of breast cancer cells, including migration, invasion, clonogenicity, cancer stem cell load and *in vivo* tumor growth and metastasis.

## Results

### Shk inhibits cancer hallmarks in breast cancer cell lines and primary cells

**Figure 1:**
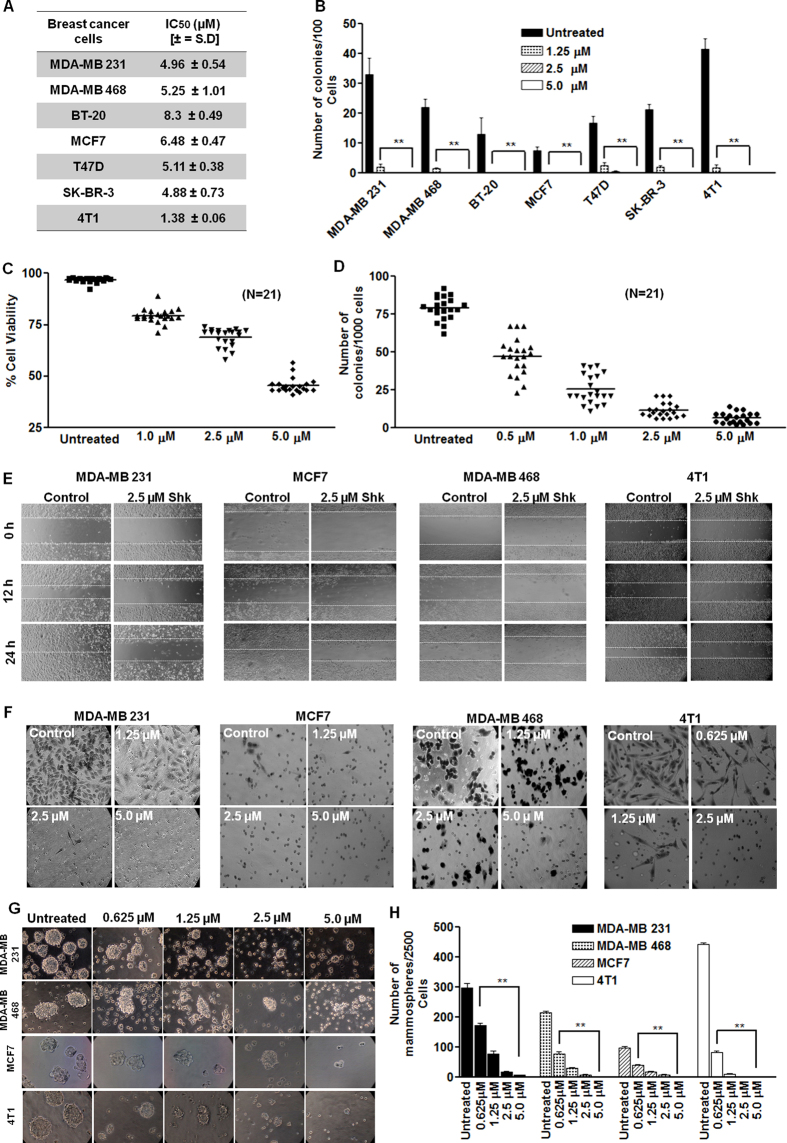
Shk inhibits multiple cancer hallmarks (**A**) The IC_50_ of Shk for indicated breast cancer cells after 24 h of treatment. (**B**) Effect of Shk on clonogenic potential of indicated breast cancer cells. (**C**) Effect of Shk on cell viability of patient derived primary breast cancer cells. (N = 21). (**D**) Effect of Shk on clonogenic potential of patient derived primary breast cancer cells. (**E**) Effect of 2.5 μM Shk on migratory potential of indicated human breast cancer cells. The wound healing in presence and absence of Shk photographed at 0, 12 and 24 h (**F**) Effect of indicated doses of Shk on invasive potential of different breast cancer cells. Cells are allowed to invade for 24 h across matrigel coated trans-well inserts. Invaded cells were fixed, stained and photographed (20X magnification). (**G**) Representative pictures of mammospheres derived from breast cancer cells in presence and absence of the different doses of Shk (20X magnification). (**H**) Effect of indicated doses of Shk on mammosphere forming potential of breast cancer cells. Data are expressed as a fold change relative to the DMSO-treated (Untreated) cells. Data are shown as the mean ±SD. (^**^) p < 0.01.

We first examined the effect of Shk on various cancer hallmark capabilities (proliferation, invasion, migration, colony and mammosphere forming potential) in breast cancer cells. MTT assay was used to find out effect of Shk on viability of breast cancer cells. Semi-confluent cultures were exposed to various concentrations of Shk for 24 h. Shk showed specific anti-breast cancer activity with IC_50_ values ranging from 1.38 μM to 8.3 μM in MDA-MB 231, MDA-MB 468, BT-20, MCF7, T47D, SK-BR-3 and 4T1 cells (Fig. 1A). Whereas the IC_50_ values in non-cancerous HEK-293 and human PBMCs were significantly higher indicating that it is relatively safe for normal cells (Fig. S1A). Shk was found to induce necroptotic cell death consistent with previous reports (Fig. S1B). Treatment of breast cancer cells for 24 h with 1.25 μM, 2.5 μM and 5.0 μM of Shk significantly reduced their colony forming potential (Fig. 1B). To check the effect of Shk on the heterogeneous cancer cell population, we tested it on patient derived primary breast cancer cells. Shk reduced the viability and colony forming potential of primary breast cancer cells in dose dependent manner (Fig. 1C,D). Further we checked its effects on migration and invasion of breast cancer cells. Shk (2.5 μM) significantly inhibited the migration of MDA-MB 231, MDA-MB 468, MCF7 and 4T1 cells (Fig. 1E). It also inhibited the cell invasion in dose dependent manner (Fig. 1F and S1C, S1D, S1E, S1F). We further examined its effect on mammosphere formation. MDA-MB 231, MDA-MB 468, MCF7 and 4T1 cell mammosphere cultures were grown in presence or absence of 1.25 μM, 2.5 μM and 5.0 μM Shk for 24 h. After 8 days of culture, a dose dependent decrease in the mammosphere forming potential of these cells was observed (Figs. 1G,H). Collectively, these results indicated that Shk effectively inhibits the various hallmarks associated with aggressive breast cancer.

### Shk reduces cancer stem cell load in breast cancer

**Figure 2:**
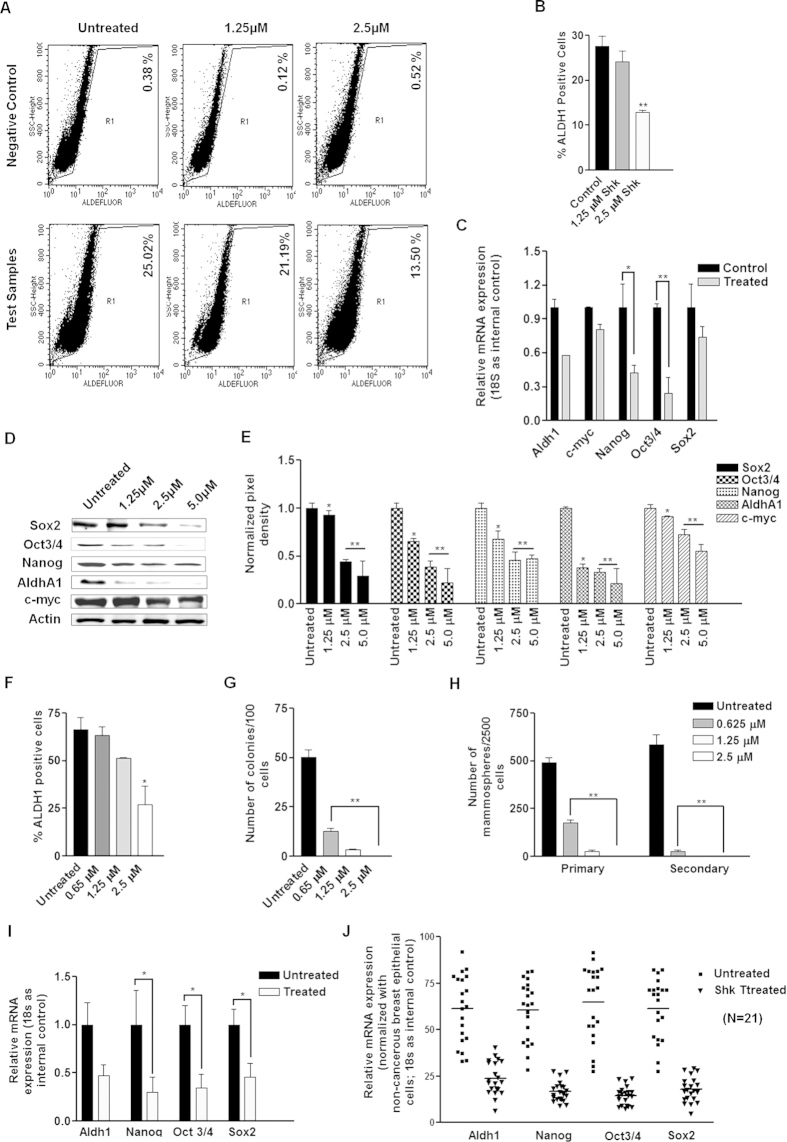
Shk decreases stem cell load in breast cancer cells and enriched CD44+,CD24−/low breast cancer stem cells. (**A**) MDA-MB 231 cells were treated with indicated doses of Shk for 24 h and ALDH1 expression was measured through flow-cytometry using Aldefluor assay. Values mentioned along with the dot plots indicate percentage of ALDH1+ population; (**B**) bar graph represents the average of three independent experiments. (**C**) Gene expression of indicated CSC markers in the MDA-MB 231 cells treated either with DMSO or 2.5 μM Shk for 24 h. Data are expressed as a fold change relative to the untreated (DMSO treated) control. (**D,E**) MDA-MB-231 cells were treated with increasing concentrations of Shk for 24 h and western blot was done for various cancer stem cell marker proteins as indicated. The full size blots corresponding to the cropped blot images are given in Fig. S10. Bar graph represents the normalized densitometric quantitation of western blot band intensities to β-actin band intensities. (**F–H**) Bar graphs represents effect of indicated doses of Shk on cancer stem cell load (ALDH1 positivity), clonogenicity and mammosphere forming potential of CD44+ CD24−/low MCF7 breast cancer cells. (**I**) Gene expression of the indicated CSC markers in CD44+ CD24−/low MCF7 cells treated with either DMSO (untreated control) or 2.5 μM Shk (treated) for 24 h. (**J**) Gene expression of indicated CSC markers in patient derived breast cancer cells treated either with DMSO or 2.5 μM Shk for 24 h. Data are expressed as a fold change relative to the untreated (DMSO treated) control. Data are shown as the mean ±SD. (*) p < 0.05, (^**^) p < 0.01 and (^***^) p < 0.001.

As Shk exhibited strong anti-mammosphere forming potential; therefore it was further examined for its anti-cancer stem cell (CSC) properties. Cancer stem cell loads in breast cancer cells were assessed using Aldefluor assay which measures ALDH1 expression. MDA-MB 231 cells with the highest number of ALDH1+ cells were selected for further studies (Fig. S2A). We also checked the correlation between ALDH1 expression and mammosphere formation. Sorted ALDH1+ cells were subjected to mammosphere cultures. ALDH1+ cells formed highest number of mammospheres compared to ALDH1-/low and parent cell population, indicating that ALDH1+ cells are enriched in CSCs (Fig. S2B). Shk reduced the Aldefluor positive cells in MDA-MB 231 cells after 24 h of treatment (Fig. 2A,B). Next, we examined the effect of Shk on the expression of stem cell (Sox2, Oct3/4, Nanog, AldhA1 and c-Myc) and EMT (Snail, Slug, ZEB1, Twist, β-Catenin) markers, associated with the sustenance of breast CSCs. Shk (2.5 μM) treatment for 24 h reduced the expression of these markers (Fig. 2C and S2D). Shk also reduced protein expression of these markers in dose dependent manner (Fig. 2D,E and S2C).

To further confirm anti-CSC properties of Shk, we checked the effect of shikonin on the load of CD44+ CD24− breast CSCs in MCF7 cells grown on matrigel. Shikonin reduced CD44+ CD24− cell load in dose dependent manner after 24 h of treatment (Fig S2E). We also tested its effects on the enriched CSC population. CD44+ CD24− cells were enriched from MCF7 cells using MagCellect CD24− CD44+ Breast CSC Isolation Kit (Fig. S2F). Enriched CSCs formed highest number of mammosphere in comparison to parent MCF7 cell population or negatively selected CD24+ cells (Fig. S2G). Enriched CSCs were treated with indicated doses of Shk (0.625 μM, 1.25 μM and 2.5 μM) for 24 h and were either analyzed for ALDH1 positivity or subjected to colony or mammosphere formation. 2.5 μM dose of Shk reduced ALDH1+ cells by 50% and inhibited colony and mammosphere formation (Fig. S2H, 2F, 2G and 2H). Shk also reduced the mRNA expression of CSC markers in CD44+ CD24− cells and patient derived primary cancer cells (Fig. 2I,J). These results collectively indicated that Shk inhibits CSC load and associated programs in breast cancer.

### Shk is a potent inhibitor of STAT3 and poorly inhibits FAK and Src

**Figure 3:**
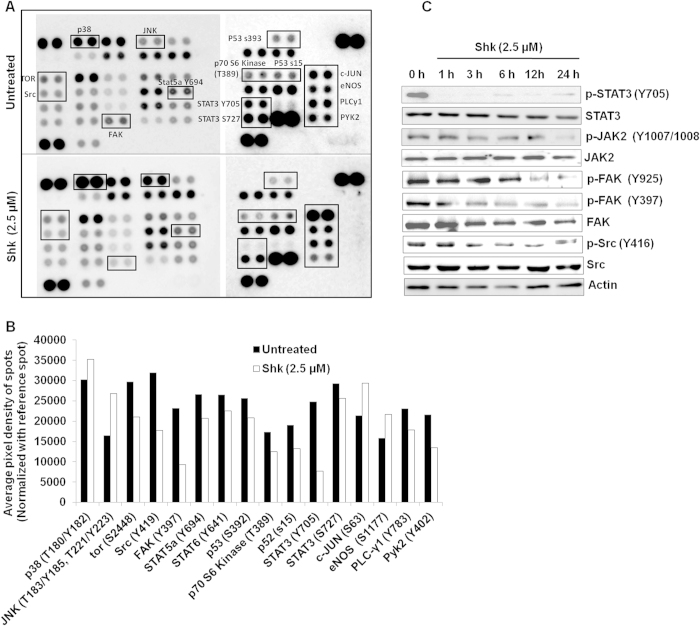
Shk inhibits STAT3, FAK and Src signaling pathways. (**A**) MDA-MB 231 cells were cultured in presence of DMSO (untreated) or 2.5 μM Shk (treated) for 6 h and cell lysates were subjected to the kinase profiling array. The array membranes were scanned and densitometry for all spots was performed. (**B**) Bar graphs represent the normalized pixel densities of some of the selected proteins differentially activated in control (DMSO treated) and Shk treated samples. (**C**) MDA-MB 231 cells were treated with DMSO or 2.5 μM Shk for indicated time intervals and western blot for various signaling molecules was performed. The full size blots corresponding to the cropped blot images are given in Fig. S10.

To identify the molecular mechanism responsible for anti-cancer properties of Shk, we used a human phospho-kinase antibody array to study a subset of phosphorylation events in MDA-MB 231 cells after 6h of treatment with 2.5 μM Shk. Amongst the 46 phospho-antibodies spotted on the array, the relative extent of phosphorylation of three proteins decreased to about ≳ 2 fold (STAT3, 3.3 fold; FAK, 2.5 fold and Src, 1.8 fold) upon Shk treatment (Fig. 3A,B). These proteins (STAT3, FAK and Src) are known to regulate CSC proliferation and self renewal^21^,^22^,^23^. Therefore, we focused on these proteins and the result of kinase-array was confirmed by western blotting. Shk effectively inhibits STAT3 at early time point (1 h) while activation of FAK and Src decreased on or after 3 h (Fig. 3C) confirming Shk as a potent inhibitor of STAT3. Shk also reduced the protein expression of STAT3, FAK and Src at 24 h (Fig. 3C).

We also observed that Shk does not inhibit JAK2 at initial time-points (Fig. 3C). This raised a possibility that Shk either regulates STAT3 independent of JAK2 or it binds directly to STAT3. To check the first probability, we activated STAT3 by treating the cells with IL6 (100 ng ml^−1^) for 1 h followed by treatment with Shk (2.5 μM) for 1 h. Both immunofluorescence and western-blotting results showed that Shk inhibited activated STAT3 without inhibiting JAK2 (Fig. S3A, S3B) confirming that Shk inhibits JAK2 mediated activation of STAT3 possibly by binding directly to STAT3. For further confirmation, we performed an *in silico* molecular docking analysis to examine binding of Shk with the STAT3 SH2 domain. In a major conformational cluster, Shk occupied Lys-707, Lys-709 and Phe-710 binding sites in the STAT3 SH2 domain similar to the STAT3 standard inhibitor S3I-201 (Fig. S3C and S3D). The binding energy of Shk to STAT3 was −4.20 kcal mol^−1^. Collectively, these results showed that Shk potently inhibits STAT3 activation and also attenuates FAK and Src activation.

### STAT3, Src and FAK are differentially expressed and activated in breast CSCs (BCSCs)

**Figure 4:**
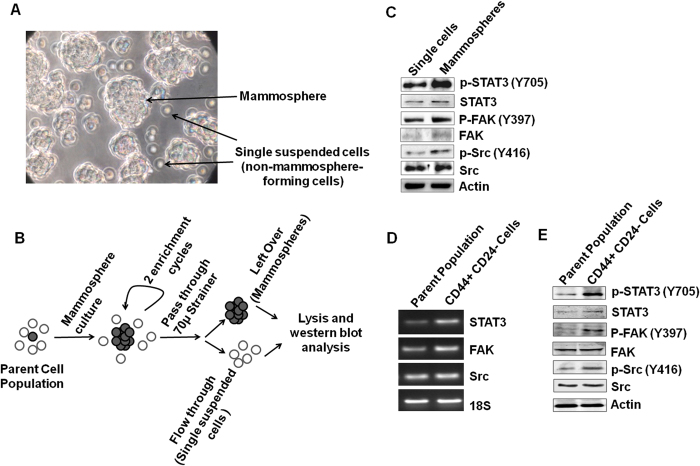
STAT3, FAK and Src are differentially activated and expressed in breast cancer cells. (**A**) Representative picture indicating mammosphere and single suspended cells. (**B**) Schematic outline of mammosphere enrichment. (**C**) Protein expression and activation of STAT3, FAK and Src was determined in single suspended cells (non-mammosphere forming cells) and mammospheres by western blot. The full size blots corresponding to the cropped blot images are given in Fig. S10. (**D**) Gene expression of STAT3, FAK and Src was determined in MCF7 parent population and CD44+ CD24−/low MCF7 cells using PCR. The full agarose gel images corresponding to the cropped images are given in Fig. S10. (**E**) Protein expression and activation of STAT3, FAK and Src was in CD44+ 24− cells and parent population.

STAT3 and FAK are known to play an important role in proliferation and self-renewal of CSCs in various cancer types including breast cancer^21^,^22^,^24^. Src also support CSC phenotype in some cancer types, but there are limited reports of its involvement in breast cancer^25^. Therefore, we checked the expression and activation of STAT3, FAK and Src in CSCs and non-CSCs. Here we used two methods to enrich the CSCs and non-CSCs. In the first method, the MDA-MB 231 cells were subjected to mammosphere formation for 96 h. After 96 h, mammosphere and non-mammosphere forming cells were clearly visible (Fig. 4A). These mammosphere and non-mammosphere forming cells were separated by using a 70 micron cell strainer. Mammospheres were subjected to two subculture cycles to enrich CSCs. With each passage, the viable single cells (non-mammosphere forming cells) and mammospheres were collected in RIPA lysis buffer and western blotting was done (Fig. 4B). We found that the activation and expression of the STAT3, FAK and Src is higher in enriched mammosphere cultures (Fig. 4C). In the second method, CD44+ CD24− cells were isolated from MCF7 cultures using MagCellect Breast CSC Isolation Kit. STAT3, FAK and Src activation and their mRNA and protein expression were assessed in enriched CSCs and were compared to parent MCF7 cell population. STAT3, FAK and Src all were differentially activated in CSCs (Fig. 4E). High mRNA as well as protein expressions of all the three genes was also observed in CSCs (Fig. 4D,E). Collectively, these results indicate that STAT3, FAK and Src are over expressed and activated in BCSCs.

### STAT3 is important for mammosphere formation and CSC programs in breast cancer

**Figure 5:**
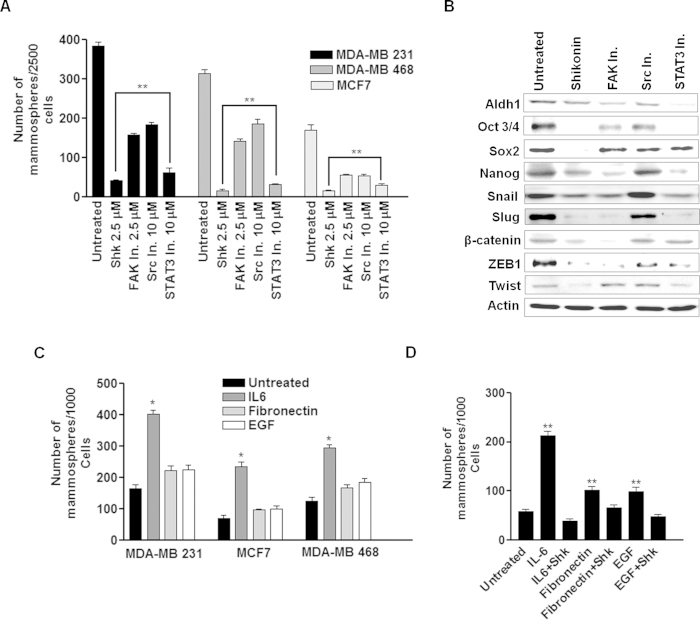
STAT3, FAK and Src activation status correlates with mammosphere forming potential in breast cancer. (**A**) Bar graph represents number of mammospheres formed from 2500 cells in presence and absence of indicated treatments. MDA-MB 231, MDA-MB 468 and MCF7 24 h mammosphere cultures were treated with Shk (2.5 μM), FAK inhibitor (FAK inhibitor 14; 2.5 μM), Src inhibitor (AZM 475271; 10 μM) and STAT3 inhibitor (WP1066; 10 μM). After 24 h, treatments were removed and cells were allowed to grow in fresh mammosphere culture media for 8 days. (**B**) Expression of various stem cell and EMT related transcription factors and markers were detected using western blotting in MDA-MB 231 cells with or without indicated treatments. The full size blots corresponding to the cropped blot images are given in Fig. S10. (**C**) MDA-MB 231, MDA-MB 468 and MCF7 cells were pre-treated with either IL6 (100 ng ml^−1^), Fibronectin (1 μg ml^−1^) or EGF (25 ng ml^−1^) for two population doublings and subjected to mammosphere formation. Bar graph represents average of three independent experiments. (**D**) MCF7 cells were pre-treated with either IL6 (100 ng ml^−1^), Fibronectin (1 μg ml^−1^) or EGF (25 ng ml^−1^) for two population doublings and subjected to mammosphere formation. After 24 h, cells were treated with DMSO (untreated) or Shk (treated) as indicated in the bar graph. Data are shown as the mean ±SD. (^*^) p < 0.05 and (^**^) p < 0.01.

As our results indicated that the expression and activation of STAT3, FAK and Src is high in BCSCs and Shk is capable of inhibiting these signaling proteins; therefore to find out functional relevance of each protein and associated effects on their pharmacological inhibition by Shk, we used specific inhibitors against these three. Effect of these inhibitors was first tested on the mammosphere forming potential of MDA-MB 231, MDA-MB 468 and MCF7 cells. A drastic reduction in the mammosphere formation was observed upon STAT3 inhibition. FAK and Src inhibition also reduced the primary and secondary mammosphere formation but STAT3 inhibition showed most potent effect (Fig. 5A and S4). Further, we also checked the effect of these inhibitors on the expression of various CSC and EMT related markers in MDA-MB 231 cells. STAT3 inhibition decreased the expression of most of the CSC and EMT markers (Fig. 5B). These two findings indicated that STAT3 inhibition is more effective in reducing mammosphere forming potential and weakens major CSC programs and the anti-CSC potential of Shk is possibly due to its strong STAT3 inhibitory effect.

To further check the involvement of these pathways in CSCs, we cultured MDA-MB 231, MDA-MB 468 and MCF7 cells in the presence of either IL6 (100ng ml^−1^), EGF (25 ng ml^−1^) or Fibronectin (1 μg ml^−1^) coated surface for two population doublings. Cells were then subjected to mammosphere formation. In IL6 pre-treated cultures, there was a sharp rise in mammosphere formation, indicating that the STAT3 activation shifts CSC and non-CSC dynamics towards CSCs (Fig. 5C). IL6 is previously known to induce the conversion of non-CSC to CSC via STAT3 activation^26^. In MCF7 cells, mammosphere forming potential after IL6 pre-treatment increased nearly by three fold. Therefore, we further checked the effectiveness of Shk on mammosphere forming potential in pre-treated MCF7 cells. It was found that Shk inhibits mammosphere formation most effectively in IL6 pre-treated cultures (Fig. 5D). However, in EGF and Fibronectin pre-treated cultures, Shk was relatively less effective. This was possibly due to its weak FAK and Src inhibitory potential. Collectively, these results illustrated that STAT3 activation is significantly correlated with the mammosphere forming potential of breast cancer cells and its inhibition by a standard inhibitor or Shk potently reduce the mammosphere formation.

### Shk inhibit CSCs load by disrupting the STAT3-Oct3/4 axis

**Figure 6:**
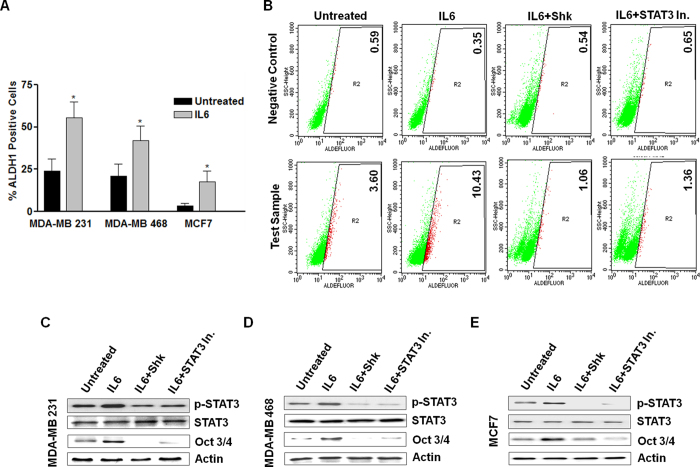
STAT3 activation status and its effect on cancer stem cell load (**A**) MDA-MB 231, MDA-MB 468 and MCF7 cells were grown with or without IL6 (100 ng ml^−1^) for two population doublings and analyzed for ALDH1 positivity using Aldefluor assay. (**B**) MCF7 cells were grown with or without IL6 (100 ng ml^−1^) for two population doublings and treated with DMSO (untreated) or indicated treatments (Shk 2.5 μM or STAT3 Inhibitor (WP1066) 10μM) for 24 h and ALDH1 expression was measured through flow-cytometry using Aldefluor assay. Values mentioned along with the dot plots indicate percentage of ALDH1+ population; bar graph represents the average of three independent experiments. (**C–E**) MDA-MB 231, MDA-MB 468 and MCF7 cells were cultured with IL6 (100 ng ml^−1^) and treated with DMSO or indicated treatments. STAT3 expression and STAT3 activation (Y705) was assessed using western blotting after 1 h of treatment. Oct3/4 expression was assessed after 24 h treatment. The full size blots corresponding to the cropped blot images are given in Fig. S10. Data are shown as the mean ±SD. (^*^) p < 0.05.

In breast cancer, STAT3 mediated expression of Oct3/4 is a major regulator of CSC self-renewal^26^,^27^. As we observed that both Shk and STAT3 inhibitors decreased the Oct3/4 expression (Figs. 2C and 5B), we further checked the effect of STAT3 activation on ALDH1+ CSCs and Oct3/4 expression. On IL6 pre-treatment, number of ALDH1+ cells increased in all three (MDA-MB 231, MDA-MB 468 and MCF7) cancer cells (Fig. 6A). MCF7 cells showed highest increase. Therefore, to check the effect of STAT3 inhibition on CSC load, we incubated IL6 pre-treated MCF7 cells with Shk and STAT3 inhibitor for 24 h and analyzed for ALDH1 positivity. It was observed that both Shk and STAT3 inhibitor reduced the IL6 induced ALDH1 positivity from 10% to < 2% (Fig. 6B). These results suggested that Shk induced inhibition of STAT3 and decrease in BCSC load is interlinked. We further checked the effect of STAT3 activation status on Oct3/4 expression in MDA-MB 231, MDA-MB 468 and MCF7 cells. We observed that expression of Oct3/4 increases with the increase in STAT3 activation (Fig. 6C–E).

STAT3 transcriptional activity is important in maintaining CSC programs^28^,^29^. Therefore, we also examined the effect of Shk on STAT3 promoter activity. STAT3 reporter assay was performed in presence of IL6 and Shk; it was found that Shk reduced the promoter activity of STAT3 in a dose dependent manner (Fig. S5). Collectively, these results showed that Shk mediated STAT3 inhibition are responsible for decrease in CSC load and Oct3/4 associated stem cell programs.

### Shk inhibits mammosphere formation, migration and invasion through inhibition of STAT3, FAK and Src in breast cancer cells

**Figure 7:**
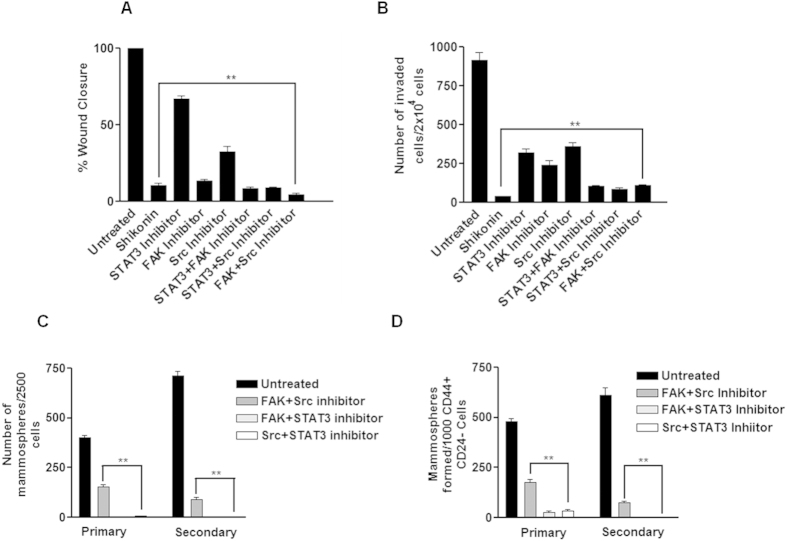
Combination of FAK, Src and STAT3 inhibitors is more potent than individual inhibition against various cancer hallmarks. (**A**) Cell migration and (**B**) cell invasion potential of MDA-MB 231 cells was assessed in the presence of Shk (2.5 μM), FAK inhibitor (FAK inhibitor 14; 2.5 μM), Src inhibitor (AZM 475271; 10 μM) and STAT3 inhibitor (WP1066; 10 μM). Various combinations of these inhibitors were also used STAT3+FAK inhibitor (WP1066; 10 μM + FAK inhibitor 14; 2.5 μM), STAT3 + Src Inhibitor (WP1066; 10 μM + AZM 475271; 10 μM) and FAK+Src Inhibitor (FAK inhibitor 14; 2.5 μM + AZM 475271; 10 μM). Cell migration and cell invasion was assessed through scratch cell migration assay and transwell invasion after 24 h of treatments. (**C,D**) Mammosphere forming potential of MDA-MB 231 cells and CD44+ CD24−/low enriched MCF7 cells was assessed in presence of similar combination of STAT3+FAK inhibitor (WP1066; 10 μM + FAK inhibitor 14; 2.5 μM), STAT3 + Src Inhibitor (WP1066; 10 μM+ AZM 475271; 10 μM) and FAK + Src Inhibitor (FAK inhibitor 14; 2.5 μM + AZM 475271; 10 μM). Cells were subjected to mammosphere cultures for 24 h and treated with the indicated inhibitors for next 24 h, followed by media change and growth of mammospheres were monitored for next 8 days. Data are shown as the mean ±SD. (^**^) p < 0.01.

As the earlier results (Fig. 1) showed that Shk inhibits cell migration and invasion in breast cancer cells, we further examined the effect of STAT3, FAK and Src inhibitors on cell migration and invasion in MDA-MB 231 cells. It was found that STAT3 inhibitor poorly inhibits cell migration while both Src and FAK inhibitors were effective in reducing cell migration (Fig. 7A). All the three inhibitors decreased the cell invasion and MMP9 expression significantly (Fig. 7B and S6). It was also observed that effect of all these inhibitors, except STAT3 inhibitor on mammosphere formation and FAK inhibitor on cell migration, were not comparable to that of Shk. Shk inhibited all these properties more effectively than individual inhibition of STAT3, FAK and Src. This made us to assume that the ability of Shk to inhibit multiple signaling molecules simultaneously is the reason behind its potent anti-cancer effect. To check this notion, we combined STAT3, FAK and Src inhibitors with each other and examined the effect of combinations on invasion, migration and mammosphere forming potential in MDA-MB 231 cells. We observed further decrease in cell migration and invasion on combining STAT3 and FAK, STAT3 and Src, or FAK and Src (Figs. 7A,B). Combination of FAK and Src was not very effective in inhibiting mammosphere formation in MDA-MB 231 cells and CD44+ CD24− MCF7 CSCs. However, their combination with STAT3 decreased the mammosphere forming potential equivalent to that of Shk (Fig. 7C,D). We also compared the mammosphere forming potential of Shk with Salinomycin (another anti-CSC agent) and found that at 2.5 μM dose of Shk was almost two times more potent than Salinomycin (Fig. S7). Collectively, these results indicated that Shk inhibits multiple signaling proteins (STAT3, FAK and Src) to compromise various aggressive breast cancer hallmarks.

### Shk inhibits breast cancer growth, metastasis and decreases tumorigenicity

**Figure 8:**
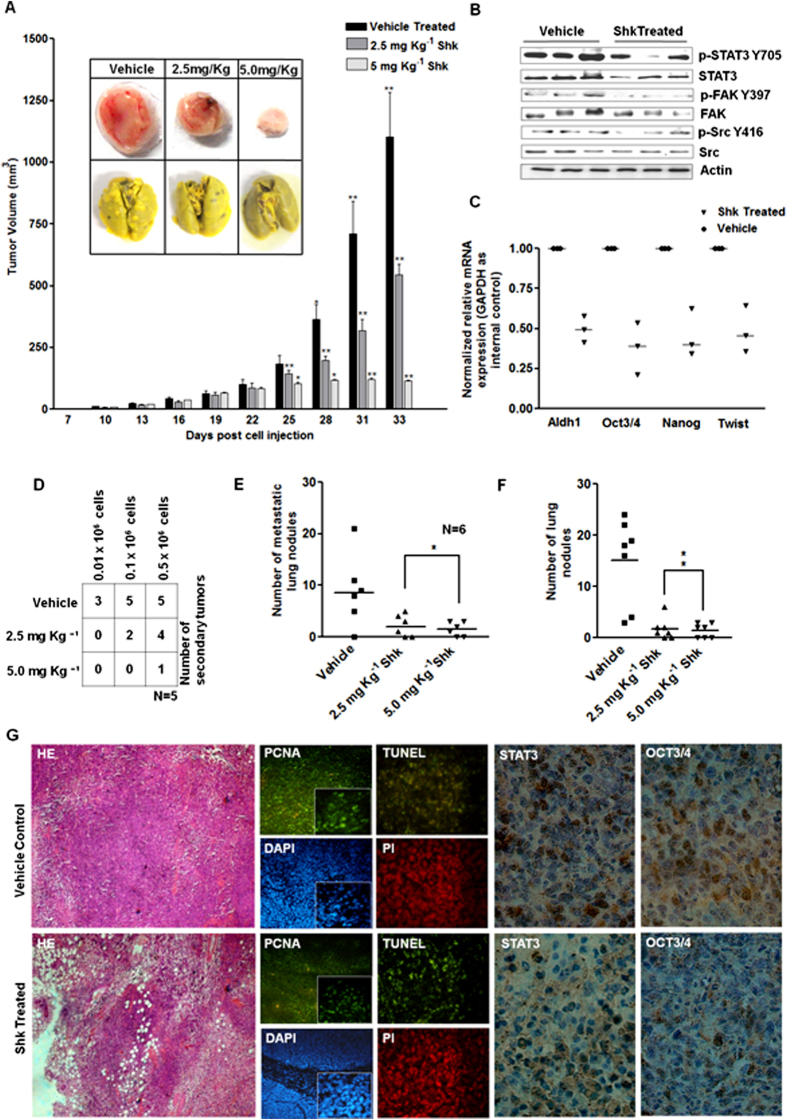
Shk inhibits breast cancer growth, tumorigenicity and metastasis *in vivo*. (**A**) Shk inhibited 4T1 tumor growth. Bar graph represents the average tumor volumes in vehicle control and Shk treated tumor bearing mice (n = 6). (^*^) p < 0.05 and (^**^) p < 0.01. Inset picture of upper panel represents tumor sizes and lower pane represents lung morphology in vehicle control and Shk treatment groups. (**B**) Western blot examination of indicated proteins for their expression and activation in vehicle control and treated tumor groups. The full size blots corresponding to the cropped blot images are given in Fig. S10. (**C**) Gene expression of stem cell and EMT markers in tumor tissues excised from the vehicle control and Shk treated groups (n = 3). (**D**) Number of secondary tumors formed after injecting indicated cell dilutions from Vehicle treated and Shk treated 4T1 tumors. (**E**) Number of lung nodules formed in mice injected with 4T1 mouse mammary tumor cells in the mammary fat pad and administered with 2.5 mg Kg^−1^ Shk or vehicle control on every alternate day for 3 weeks (n = 6). (**F**) Number of lung nodules in mice injected with 4T1 mouse mammary tumor cells through tail vein and administered with 2.5 mg Kg^−1^ Shk or vehicle control on every alternate day for 3 weeks. (n = 8) (**G**) Representative panel of the histological H&E staining, immunofluorescence staining for the STAT3, Oct3/4, cell proliferation marker PCNA and DNA damage indicator-TUNEL staining of tumor sections from vehicle and treatment groups.

To explore whether Shk may have therapeutic potential for breast cancer treatment *in vivo*, we tested Shk against 4T1-induced breast cancer syngenic mouse model. 4T1 cells (mouse breast cancer cells) are capable of growing fast and metastasize efficiently *in vivo*^30^. Prior to the *in vivo* experiments, we checked the effect of Shk on ALDH1 positivity and on activation of STAT3, FAK and Src in 4T1 cells *in vitro*. Shk effectively decreased the ALDH1+ cells and inhibited STAT3, FAK and Src in 4T1 cells *in vitro* (Fig. S8A and S8B). For *in vivo* tumor generation, 1 × 10^6^ cells were injected subcutaneously in the fourth nipple mammary fat pad of BALB/c mice. When the average size of tumors reached around 50 mm^3^, mice were divided into three groups, vehicle and two Shk treated groups each received either 2.5 mg Kg^−1^ or 5.0 mg Kg^−1^ Shk. Shk was administered via the intraperitoneal injection on every alternate day. It significantly suppressed the tumor growth in 4T1 induced syngenic mouse model (Fig. 8A). The average reduction in 4T1 tumor growth was 49.78% and 89.73% in 2.5 mg Kg^−1^ and 5.0 mg Kg^−1^ groups respectively compared with the vehicle treated group (Fig. 8A). No considerable change in body weight of the treated group animals was observed (Fig. S9A). We further examined the effect of Shk on the tumor initiating potential of breast cancer cells. 4T1 induced tumors were excised from the control and treatment groups on the second day after 4^th^ dose of Shk was administered. Tumors were dissociated; cells were allowed to adhere and then re-injected into new animals for secondary tumor formation. Growth of secondary tumors was monitored till day 15 post-reinjection. Shk treated groups showed a marked decrease in secondary tumor formation (Fig. 8D). We also observed a drastic reduction in the number of metastatic nodules in the lungs of treatment group animals (Fig. 8F). The reduction in the metastatic load was not proportional to the decrease in tumor sizes; however within the treatment group, some animals with small tumors were carrying higher number of metastatic nodules. As FAK is an important mediator of cancer metastasis and metastatic colonization, we further examined the effects of Shk on metastatic colonization. For this, 1 × 10^5^ 4T1 cells were injected to BALB/c mice through tail vein. Animals were divided into three groups, as indicated above. Shk and vehicle were administered through intraperitoneal injections at alternate days starting from the 2^nd^ day post tail vein injections till 33^rd^ day. The average reduction in total number of metastatic nodules was 88.6% - 90.5% in Shk treated mice compared to vehicle control (Fig. 8F). An inset picture (Fig. 8A lower panel) represents lung morphology of vehicle control and treated groups. We further examined the activation and expression status of STAT3, FAK and Src between vehicle control and treated group tumors. There were low expression and activation of STAT3, FAK and Src in treated tumors as compared to the vehicle control (Fig. 8B,C). Similar trend was observed in ALDH1 expressions (Fig. 8B). Further, the mice tumor sections were subjected to immunohistochemistry, immunofluorescence and hematoxylin and eosin (H&E) staining to study histology and expression of key proteins being examined in this study. Fig. 8G shows representative images of H&E staining, proliferating cell nuclear antigen (PCNA), terminal deoxynucleotidyl transferase dUTP nick end labeling (TUNEL), STAT3 and Oct3/4 immunostaining. PCNA expression was low while TUNEL positive cells were high in tumor tissues of Shk treated groups. STAT3 and Oct3/4 expression was low in Shk treated groups. These results collectively demonstrated that Shk modulates the expression and activation of STAT3, FAK and Src *in vivo* and is effective in suppressing tumorigenic potential and metastasis in syngenic mouse model.

## Discussion

Recent studies have shown that aggressiveness, therapy resistance and disease relapse in breast cancer is attributed to a small population of CSCs involved in continuous self-renewal and differentiation through signaling pathways similar to that of the normal stem cells^31^. Therapeutic targeting of CSCs therefore, has profound clinical implications for cancer treatment^31^. Recent studies indicated that therapies / agents targeting both differentiated cancer cells and CSCs may possibly have significant therapeutic advantages^32^. Therefore, it is imperative to look for novel therapeutic agents with lesser side effects urgently for effective targeting of CSCs. In search of novel, nontoxic anti-CSC agents, attention has been focused on natural agents in recent times^33^,^34^. In this study, we have used a natural napthoquinone compound, Shk with established antitumorigenic, favorable pharmacokinetic and toxicity profiles and report for the first time its potent anti-CSC properties. Shk significantly inhibits breast cancer cell proliferation *in vitro*, *ex vivo* and *in vivo*. It decreases the cell migration and invasion of breast cancer cells *in vivo*, as well as inhibits tumorigenicity, metastasis and metastatic colonization in a syngenic mouse model of breast cancer *in vivo.* These finding suggest a strong potential of Shk in breast cancer therapy.

We assessed the effect of Shk on the CSC load in breast cancer cells through various functional assays (tumorsphere *in vitro* and syngenic mouse model of breast cancer *in vivo*) and quantification of specific stem cell markers. In breast cancer, CD44+ CD24− cells and ALDH1+ cells are considered to be BCSCs^21^,^25^. Shk significantly decreased the mammosphere formation (Fig. 1H, S1G and 2H), ALDH1+ cell and CD44+ CD24− cell loads *in vitro* (Fig. 2B, S2E and S2H). It also reduced the expression of CSC markers (Oct3/4, Sox2, Nanog, c-Myc and Aldh1) *in vivo* and *in vitro* (Fig. 2C,D, S2C and S2D). These genes are known to regulate stem cell programs and in cancer, they are established promoters and regulators of CSC phenotype^35^,^36^,^37^,^38^,^39^,^40^. Decrease in the expression of these genes on Shk treatment indicates its potential to suppress CSC programs. Tumor initiating potential (tumorigenicity) is the bona fide measure of CSCs. Reduction in the tumorigenic potential of cells isolated form Shk treated tumors indicates *in vivo* anti-CSC effects of Shk.

We further demonstrated that Shk is a potent inhibitor of STAT3 and it also inhibits FAK and Src (Fig. 3A–C). Its STAT3 inhibitory property was found to be responsible for its anti-CSC effects (Figs. 6B and 7B). STAT3 and FAK inhibitors are previously known to compromise CSC growth^41^,^42^. Here, we found that pharmacological inhibition of STAT3 was more effective in compromising CSC load than FAK and Src inhibitions (Fig. 5A). STAT3 activation through IL6 increases mammosphere formation more significantly than Src and FAK activation through EGF and Fibronectin (Fig. 5C). This indicates that IL6-STAT3 axis is a key regulator of BCSC dynamics.

In recent years, various studies had indicated that CSCs remains in dynamic equilibrium with non-CSCs^26^,^43^. Under suitable microenvironment non-CSCs may convert into CSCs and *vice-versa*^44^. The IL6/STAT3/Oct3/4 axis plays an important role in conversion of non-CSCs to CSCs^27^. IL6 through its receptor activates STAT3 which regulates expression of Oct3/4, a major reprogramming factor known to induce expression of various stem cell associated genes and suppression of lineage commitment genes^45^. Various studies suggest a direct co-relation in expression of both Oct3/4 and STAT3 in cancers^27^,^46^. Consistent with these studies, we found that Shk decreased the STAT3 activation, expression and Oct3/4 expression both *in vitro* and *in vivo*. (Figs. 2D,5B and 8B) STAT3 activation not only converts non-CSCs to CSCs, it also helps in self-renewal of CSCs^47^. Cytokines and growth factor induced signaling promote self-renewal via STAT3 activation and help in maintaining CSC load in cancer tissue^47^,^48^. Shk also restricted the increase in CSC load in MCF7 cells on IL6 treatment, indicating that it is able to restrict the native and induced stemness in breast cancer. In addition to IL6 and other growth factor signaling, EMT is also known to endow stem cell like properties in cancer^49^. Shk treatment also reduced the expression of EMT markers. Among the STAT3, FAK and Src inhibitors, STAT3 inhibition (Fig. 5B) reduced the expression of Snail, Slug, Zeb1 and Twist1 most effectively. This further supports the efficacy of Shk and STAT3 inhibition against stem cell and EMT programs in breast cancer.

Apart from STAT3, FAK and Src also support stem cell like properties in cancer^22^,^24^,^25^. FAK is known to promote tumor growth and metastasis^50^,^51^. Shk inhibit the FAK and Src activation. Inhibition of FAK and Src using specific inhibitors reduced the mammosphere forming potential in breast cancer cells (Fig. 5A). This suggested that apart from known anti-CSC potential of STAT3 and FAK inhibitions, Src ablation could also serve as an anti-CSC therapeutic strategy. FAK and Src are widely known to regulate cell migration and invasion in various cancer types and their suppression decreases migratory and invasive potential^51^,^52^,^53^. Our results indicate that FAK and Src inhibitions were effective in reducing cell migration and invasion (Fig. 7A,B). Inhibition of STAT3, FAK and Src reduced the expression of MMP9 which is essential for cell migration and invasion^53^ (Fig. S6). When STAT3 and FAK or Src were inhibited simultaneously using specific inhibitors, it reduced the invasion, migration and mammosphere formation more efficiently than individual inhibitions (Fig. 7A,B). This suggests that inhibition of FAK or Src along with STAT3 is of greater therapeutic importance rather than individual inhibitions. In line with these observations, our studies confirmed the CSC inhibitory effect of Shk through suppression of these multiple targets thereby underscoring its translational relevance.

We further validated the effects of Shk on metastatic progression using a 4T1 based syngenic mouse model. 4T1 cells metastasize efficiently to sites affected commonly in human breast cancers^54^,^55^. In current study, we also observed that 4T1 cells metastasize very efficiently to lungs and treatment with Shk reduced the tumor growth, metastatic progression and metastatic colonization (Figs. 8A, 8E and 8F). STAT3 or FAK activation both are previously known to influence the tumor growth and metastasis in syngenic 4T1 mouse model^56^,^57^. We also found that Shk decreases the STAT3, FAK and Src activation and expression *in vivo* (Fig. 8B). Expression of CSC markers also reduced in tumor tissues derived from Shk treated animals (Fig. 8C). These findings indicate that the Shk inhibited CSC programs *in vivo* and reduces tumor growth and metastasis by inhibiting STAT3, FAK and Src.

In conclusion, we showed for the first time that the natural napthoquinone compound Shikonin inhibited STAT3 /FAK /Src and induced the suppression of stem cell load *in vitro* and metastasis and tumorigenicity *in vivo*. These findings suggested that Shk has promising anti-cancer and anti-CSC effects in breast cancer cells. These findings hence, provide a strong rationale for investigating the chemoprevention property of Shk with special emphasis on reduction of CSC load in clinical trials.

## Materials and Methods

### Reagents and antibodies

Shikonin and STAT3 inhibitor (WP1066) were purchased from Calbiochem; FAK inhibitor 14 and Src inhibitor (AZM 475271) from Tocris, and Salinomycin was purchased from Sigma. 10 mM Stock solution for all were prepared in DMSO, aliquoted and stored at −20 °C.

IL6 (human), IL6 (mouse), EGF (human) and EGF (mouse) were purchased from Peprotech (NJ, USA). Fibronectin from Calbiochem and B27 was purchased from invitrogen.

Cell invasion transwell inserts and matrigel were purchased from BD. Propidium iodide (PI), DAPI and Giemsa were purchased from Sigma.

Antibodies for Snail, Slug, ZEB1, β-catenin, MMP9, p-FAK(Y397), p-FAK(Y925), FAK, ALDH1A1, c-Myc, Nanog, p-STAT3(Y705), STAT3, p-JAK2(Y1007/1008), JAK2, p-Src(Y416), Src and Actin were purchased from CST; antibodies for Twist1, Sox2, Oct3/4, and PCNA were purchased from Santa Cruz Biotechnology Inc. p-JAK2(Y1007/1008) antibody for immunofluorescence analysis was purchased from Abcam.

### Cell culture

The human breast cancer cells MDA-MB 468, BT20, MDA-MB 231, MCF7, T47D, SK-BR-3, HEK-293 and mouse 4T1 breast cancer cells were obtained from the American Type Culture Collection. MDA-MB 468 and HEK-293 cells were maintained in DMEM (Sigma) supplemented with 2 μM L-glutamine (Sigma); BT20 cells were maintained in EMEM (Sigma) supplemented with 1 mM Sodium Pyurvate (Sigma); MDA-MB 231, MCF7, T47D, 4T1 and PBMCs were maintained in RPMI 1640 cell culture media (Sigma); SK-BR-3 cells were maintained in DMEM/F12 cell culture media (Sigma). All culture media were supplemented with 10% FBS and 1% Penicillin-Streptomycin (P+S) and cultures were grown at 37 °C with 5% CO_2._

### Isolation and culture of primary breast epithelial cells

Breast cancer tissues were obtained from 21 patients affected by early breast cancer after informed consent (median age 57.8 years, range, 41.1 – 61.5) undergoing surgery. Samples were collected and processed according to the approved protocol of CSIR-CDRI institutional ethics committee (human research). Tumor specimens were processed within 1 h of surgery. Brief method is discussed in supplementary materials and methods section.

### Cell viability detection

Cell viability of breast cancer cells were assessed by either using MTT assay (sigma) or ATPLite 1step assay system (Perkin Elmer) according manufacturer instructions. Details of both assays are given in supplementary materials and methods.

### Clonogenicity assay

Cells seeded at low density in a 6-well plate were treated with suitable compounds or inhibitors in complete media. After 24 h media was replaced with fresh 2 ml media and cells were allowed to grow for next 8 days, colonies containing > 50 normal-appearing cells were counted.

### Scratch-migration assay

Cells were seeded in a 6 well plate and grown till they reach 90–100% confluency. A scratch was made through the cell layer using a sterile micropipette tip. After washing with PBS, complete medium was added with and without treatments. Wounded areas were photographed under a light microscope at 4X objective at 0, 12 and 24 h.

### Invasion assay

Matrigel invasion assay was performed by using matrigel coated Boyden chambers with 8 μM pore filter inserts in 24-well plates (BD). Briefly, 2 × 10^4^ cells in 5% FBS supplemented culture medium were added to the inserts and 20% FBS supplemented media was added to the respective well. Treatments were added to both upper as well as lower chambers according to the experimental requirement. After 24 h, the non-invaded cells were gently removed with cotton swabs; invaded cells were first fixed in 4% formaldehyde and then stained with Giemsa, air-dried, counted and photographed.

### Mammosphere cultures

Single cells were plated in ultralow attachment plates (Corning) in low densities. Cells were grown in a serum-free DMEM/F12 (Sigma) supplemented with 2% B27 (Invitrogen), 20 ng/ml EGF (Peprotech) and 1% P+S. Media was changed every third day.

### Aldefluor assay and separation of the ALDH1+ cell population by FACS

Aldehyde dehydrogenase 1 (ALDH1) enzyme activity in viable cells was determined using Aldefluor® assay (Stem Cell Technologies) according to the manufacturer’s instructions. 1 × 10^6^/ml cells were suspended in Aldefluor® assay buffer containing ALDH substrate and incubated for 45 min at 37 °C. Cells incubated with Aldefluor® substrate diethylaminobenzaldehyde (DEAB) were used as reference control. The brightly fluorescent ALDH1-expressing cells (ALDH1high) were detected in the green fluorescence channel (520–540 nm) of FACSCalibur (BD Biosciences). Nonviable cells were excluded using PI (2 μg ml^−1^). FACSAria (BD Biosciences)was used for sorting ALDH1+ cells.

### CD44+ CD24−/low cell enrichment

CD44+ CD24−/low cell enrichment was done using MagCellect CD24− CD44+ Breast Cancer Stem Cell Isolation Kit (R&D System) according to the manufacturer’s instructions. Briefly, CD24+ cells were initially tagged and removed magnetically. CD44+ cells were subsequently isolated magnetically from the CD24– population by positive selection using a biotinylated human CD44 antibody and streptavidin-conjugated magnetic beads. The efficiency of enrichment was assessed by staining the recovered cells with fluorochrome-conjugated anti-human CD24 and CD44 antibodies and flow-cytometry analysis.

### Reverse transcription PCR, quantitative PCR and quantitative real-time RT-PCR

Total RNA was extracted using Trizol (Invitrogen), before reverse transcription by using superscript III (Invitrogen). Obtained cDNA was amplified using specific primers. Quantitative real-time PCR was performed in Light Cycler 480 Instrument II (Roche) using SYBR Premix Ex Taq master mix (Takara Biotechnology). Relative mRNA was determined by using the formula 2^−ΔCT^ (CT; cycle threshold) where ΔCT = CT (target gene) - CT (18S or GAPDH). Primer sequences are given in supplemental materials.

### Western blotting

Whole cell lysate was prepared by scrapping the cells in ice-cold RIPA lysis buffer supplemented with protease and phosphatase inhibitor cocktail (Thermo Scientific). 40 ug of protein was resolved on SDS-PAGE gel, transferred to nitrocellulose membrane (Millipore). Immunodetection was done using enhanced chemiluminescence (Millipore) according to manufacturer’s instruction.

### Human Phospho-Kinase Antibody Array

Human Phospho-Kinase Antibody Array (R&D Systems) was performed as per the manufacturer’s instruction. Briefly, the cell lysates (800 μg) were mixed with array buffer and incubated with pre-blocked array membrane at 4 ^o^C for overnight. Membranes were then washed and incubated with primary antibody cocktail for 2 h, followed by washing and incubated with secondary antibody for 30 min. Membranes were washed again and subjected to chemiluminescent detection.

### Immunofluorescence (IF) and Immuno-histochemistry (IHC)

2 × 10^4^ cells were seeded in 8 well culture slides (Corning) and treated with indicated doses of Shk for indicated time interval. Post-treatment cells were washed with PBS, fixed with ice-cold 4% paraformaldehyde for 15 minutes. Alternatively for IHC, mouse tumor tissues were embedded in paraffin and sliced into 6 mM-thick sections. One batch of all the sections was used for hematoxylin and eosin staining; other batches were used for IF analysis. For this purpose, sections were re-hydrated, subjected to antigen-retrieval in citrate buffer 0.01 M, pH 6 at 95 °C for 30 min. Further, formaldehyde fixed cell or rehydrated tissue section slides were blocked with 5% BSA. Samples were probed with primary antibodies (1:100) for 8–10 h followed by washing and re-probing with Alexa fluor (1:500) conjugated secondary antibodies. Slides were washed again and cells were counterstained with DAPI. Staining was visualized by epifluorescence (Olympus, BX60).

For HE staining rehydrated section were stained with Hematoxylin and Eosin, dehydrated, mounted with DPX (Sigma) and visualized under microscope.

### TUNEL Assay

Rehydrated sections were immersed in 0.85% NaCl, fixed in 4% formaldehyde for 25 minutes at 4 °C, followed by PBS washing and treated with 20 μg ml^−1^ Proteinase K for 10 minutes. Slides were washed, fixed with formaldehyde and equilibrated for 5–10 min. Following equilibration cells were labeled with TdT reaction mix (Qiagen) for 60 minutes at 37 °C in a humidified chamber. Reaction was stopped using stop solution; slides were washed three times, counterstained with PI and mounted with Citifluor AF1 mountant media (Ted Pella).

### *In Vivo* Tumor Generation and metastasis

Animal studies were conducted in accordance with the principles and standard procedures approved by IEAC at the CSIR-Central Drug Research Institute. 4T1 cells (1 × 10^6^) were injected subcutaneously into the mammary fat pad of 4 to 5 week-old female, BALB/c mice. After the tumors reached 50 mm^3^ in size, mice were divided into control and treatment groups. Control group was administered with vehicle (10% DMSO and 20% ethanol in PBS) and treatment group with different doses of Shk on alternate days for 3 weeks. Tumor dimensions (Length and Width) were measured using a caliper. Tumor volumes were calculated by the formula V = 1/2 (L × W^2^).

For 4T1 syngenic breast cancer metastasis model, 5 × 10^5^ cells were injected through tail vein of 7 to 9 week old female BALB/c mice. From the second day of injections, mice were injected with vehicle or Shk 2.5 mg Kg^−1^ on alternate days for 3 weeks.

To collect tumor and lungs, mouse were euthanized and organs were resected for further analysis. Tumor burden in the lung was quantified by manually counting nodules visible on the lung surface.

### Statistical analysis

All assays, with established cell lines were repeated three times. Assays with cells from patient tumors were repeated only once due to limitation of sample availability. The differences between control and compound treated samples were determined using statistical software (GraphPad Instat software package). Comparisons among different groups were performed by analysis of variance using one-way ANOVA. Significant differences between control and treatment groups were analyzed by Dunnett’s Multiple Comparison Test.

## Author Contributions

R.Th. and D.P.M. designed the study, analyzed the data and prepared the draft of manuscript. R.Th. carried out *in vito* and *in vivo* experiments. R.Tr. and N.R. helped in *in vivo* experiments. M.S. carried out the *in silico* studies. All authors reviewed the manuscript.

## Additional Information

**How to cite this article**: Thakur, R. *et al*. Inhibition of STAT3, FAK and Src mediated signaling reduces cancer stem cell load, tumorigenic potential and metastasis in breast cancer. *Sci. Rep.* **5**, 10194; doi: 10.1038/srep10194 (2015).

## Supplementary Material

Supplementary Information
