# Supplementary material for: Inhibition of STAT3, FAK and Src mediated signaling reduces cancer stem cell load, tumorigenic potential and metastasis in breast cancer
Source: Sci Rep. 2015 May 14;5:10194. doi: 10.1038/srep10194 (PMC4431480; doi:10.1038/srep10194)
Supplement: Supplementary Information [file srep10194-s1.pdf]

The natural compound Shikonin reduces cancer cancer stem cell load, tumorigenic potential and metastasis in breast cancer

Ravi Thakur[1], Rachana Trivedi[1], Namrata Rastogi[1], Manisha Singh[1], Durga Prasad Mishra[1*]

[1]Cell Death Research Laboratory, Endocrinology Division, CSIR-CDRI, Lucknow, INDIA

*Corresponding Author: D.P. Mishra, Cell Death Research Laboratory, Endocrinology Division, CSIR-Central Drug Research Institute, Lucknow-226031, INDIA. Tel: +91-522-2612411-18 Extn. 4387; Fax: +91-522-2623405; E-mail: dpm@cdri.res.in

Supplementary Information:

Part 1- Supplementary Figures 1-9

Part 2- Supplementary Material and Methods

Part 1: Supplementary Figures

Supplementary Figure 1

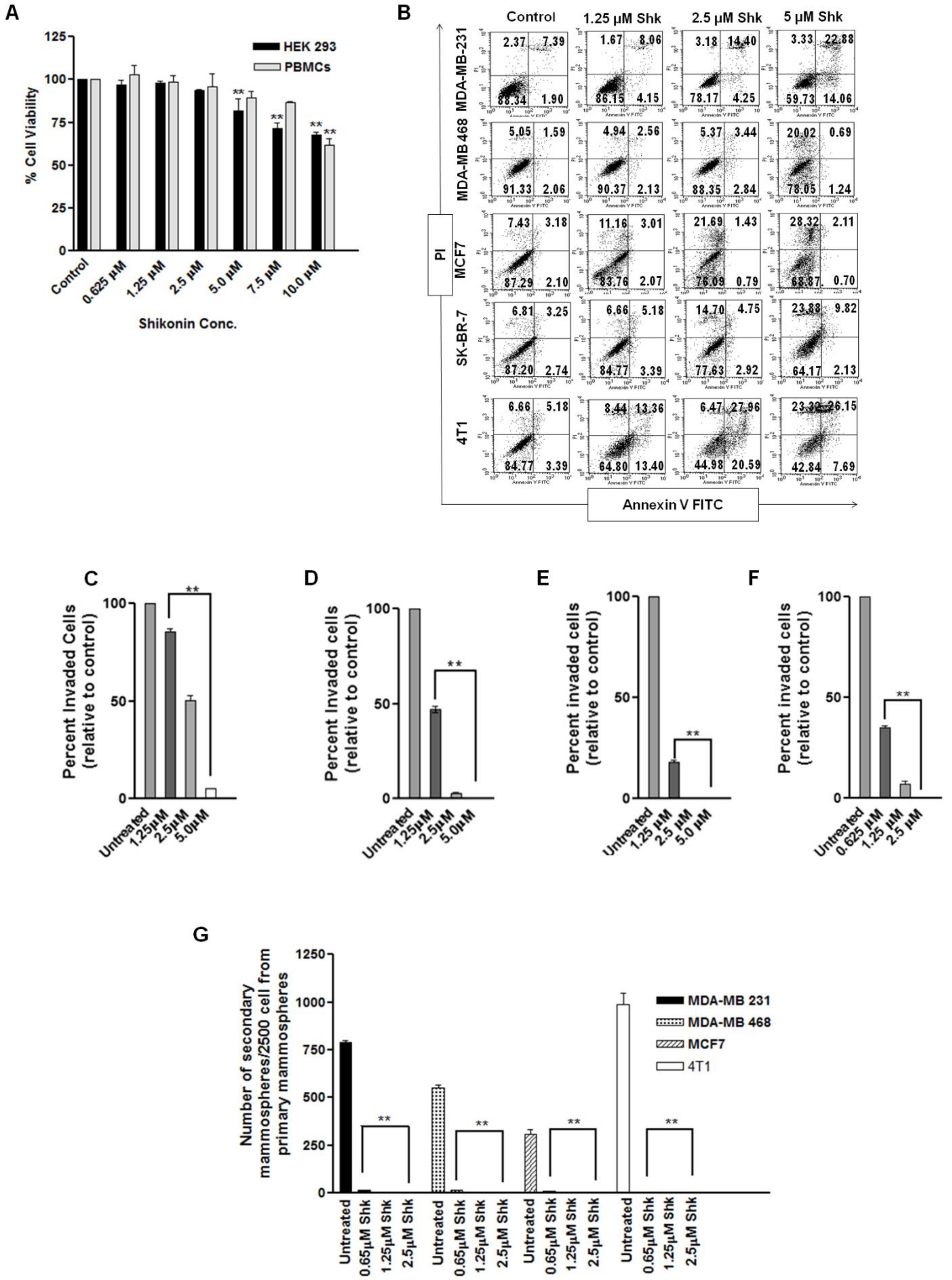

Supplementary Figure 1. Effect of Shk on normal and breast cancer cells. (A) Cell proliferation of normal control cell lines HEK293 and human PBMCs were accessed using variable doses of Shk using MTT assay. **(B)** Shk induces necroptotic cell death in breast cancer cells. Cell death induced by Shk was measured in breast cancer cells using Annexin V-alexa flour 488 and PI labeling of cells and analyzed by flow cytometry. Values are the percent of cells in each quadrant. **(C, D, E, F)** Bar graphs represent the quantization of the effect of indicated doses of Shk on invasive potential of MDA-MB231, MDA-MB 468, MCF7 and 4T1 cells respectively. Invasion was measured using matrigel coated trans-well inserts. **(G)** Bar graph represents the number of secondary mammospheres formed from the primary mammosphere cultures treated with indicated doses of Shk for 24 h. Data are shown as the mean ±SD of three independent experiments. (**) $p < 0.01$.

Supplementary Figure 2

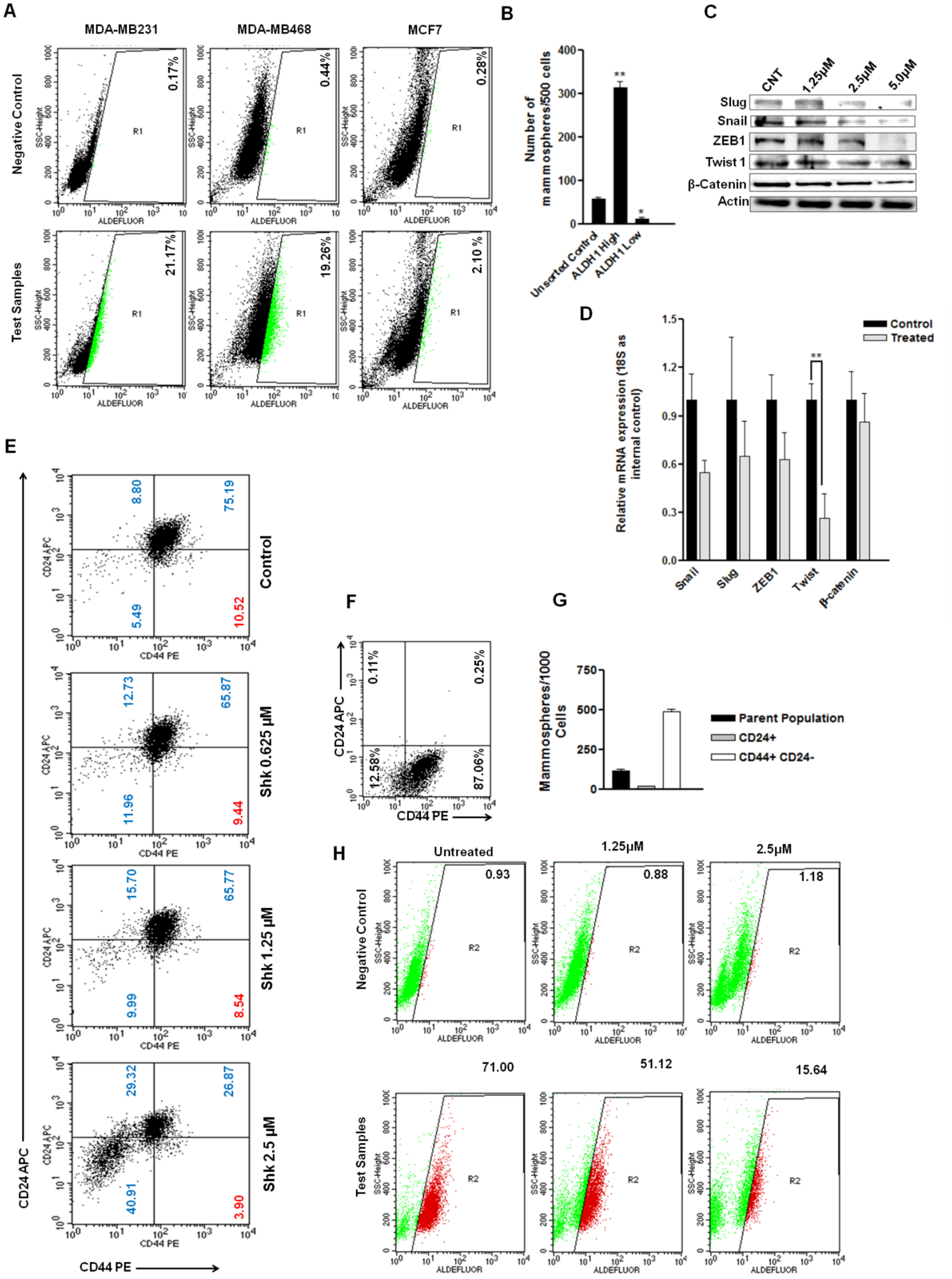

Supplementary Figure 2. Effect of shikonin on breast-CSC load and CSC associated markers (A) Comparison of ALDH1 positive population in MDA-MB231, MADA-MB 468 and MCF7 cells by aldeflour assay; Upper panel represents DEAB treated negative controls while lower panel represents test cell samples. **(B)** MDAM-MB 231 cell labeled with aldeflour, were subjected to cell sorting. ALDH1 high and ALDH1 low cell were isolated, counted and subjected to mammosphere formation; (*) $p < 0.05$ and (**) $p < 0.01$. **(C)** MDA-MB 231 cells were treated with indicated doses of Shk and protein expression of mesenchymal markers were detected by western blot. **(D)** mRNA expressions of various EMT associated genes. MDA-MB231 cells were treated with solvent (DMSO) or Shk (2.5μM) for 24 hours. Total RNA was isolated and subjected to quantitative real-time PCR. Data are presented as fold change in mRNA levels and normalized to control treatment with solvent (DMSO) Results are compilation of three independent experiments performed in triplicates; (*) $p < 0.05$. **(E)** Effect of Shk on CD44+ CD24- cell load in MCF7 cells. **(F)** CD44+ CD24-/low cells were enriched using MagCellect CD24- CD44+ Breast Cancer Stem Cell Isolation Kit. Post enrichment cells were analyzed for CD44 and CD24 expression. The dot plot is a representative of one of the enriched MCF7 population analyzed for surface markers and indicates percentage of cells in respective quadrants. **(G)** Enriched CD44+ve CD24-ve/low MCF7 cells were counted and subjected to mammosphere formation. The bar graph represents average of three independent experiments. (*) $p < 0.05$ and (**) $p < 0.01$. **(H)** Dot plots are representative of aldefluor assay performed with CD44+ CD24-/low MCF7 cells treated with indicated doses of Shk.

Supplementary Figure 3

A

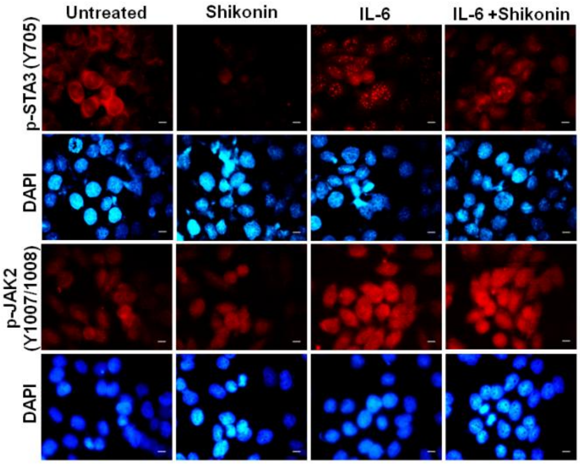

B

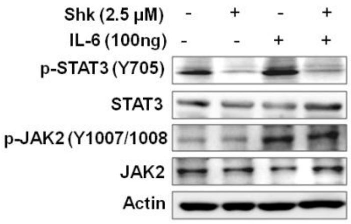

C

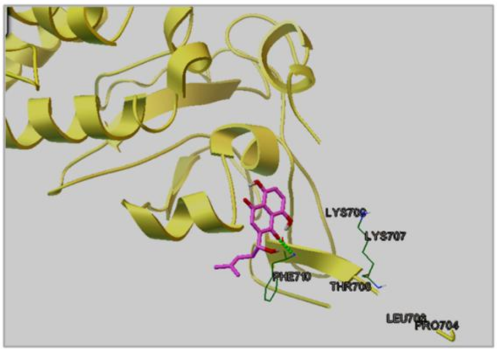

D

S.No	Ligand	Receptor	H-Bonds	Interacting residues	Docking Score (Kcal mol^{-1})
1	S3I-201	STAT3	4 (PHE710,ASN538, LYS707,LYS709)	SER649,ARG688,LYS573,LEU577, LYS685,ASN538,TYR539, LYS707,LYS709, PHE710	-4.60
2	SHK	STAT3	4 (PHE710,TRP501, LYS707,LYS709)	TRP501,THR526, LYS685,ASN538,GLU582, ASN646, LYS707,LYS709	-4.20

Supplementary Figure 3. Shikonin inhibits STAT3. (A) Immunofluoresence analysis of p-STAT3 Y705 and p-JAK2 Y1007/1008 were performed in presence and absence of Shk, IL6 or both. MDA-MB 231 cells were treated with 2.5μM of Shk, IL6 (100ng) or both **(B)** Activation status of p-STAT3 Y705 and p-JAK2 Y1007/1008 was detected using western-blot in MDA-MB231 cells after 1 hour of treatment with either Shk or 100ng IL6 or both. **(C)** Computer modelling of Shk binding to STAT3 SH2 domain. Shk is in thick stick-ball (S-B) model and in pink and red colors. **(D)** STAT3 SH2 binding properties of Shk were compared with known STAT3 inhibitor S3I-201 using molecular docking. (*) $p < 0.05$, (**) $p < 0.01$.

Supplementary Figure 4

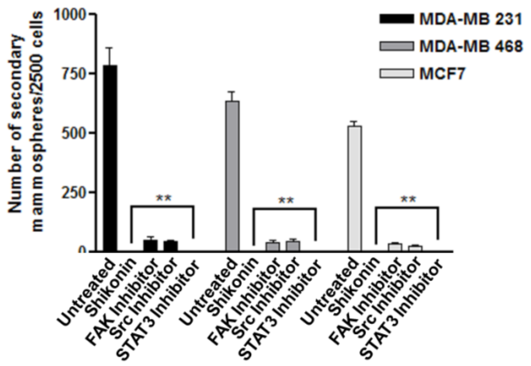

Supplementary Figure 4. Effect of FAK, Src and STAT3 inhibition on secondary mammosphere formation in breast cancer cells. The primary mammosphere cultures treated with Shk (2.5µM), FAK inhibitor (FAK inhibitor 14; 2.5µM), Src inhibitor (AZM 475271; 10µM) and STAT3 inhibitor (WP1066; 10µM) for 24 h after completion of 8 days of culture without treatment were subjected to secondary mammosphere formation. The bar graph represents the average of three independent experiments performed in duplicates. (**) $p < 0.01$.

Supplementary Figure 5

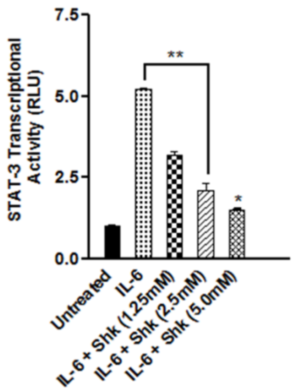

Supplementary Figure 5. Shikonin decreases STAT3 transcriptional activity. The effect of shikonin on the transcriptional activity of STAT-3 was studied using a CIGNAL Reporter Assay kit. The bar graph represents average values from three independent experiments. (*) $p < 0.05$, (**) $p < 0.01$.

Supplementary Figure 6

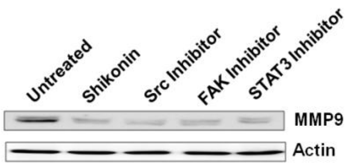

Supplementary Figure 6. FAK, Src, STAT3 and Shk independently decreases MMP9 expression. The expression of MMP9 in MDA-MB 231 cells was detected by western blotting after 24 h of Shk (2.5µM), FAK inhibitor (FAK inhibitor 14; 2.5µM), Src inhibitor (AZM 475271; 10µM) and STAT3 inhibitor (WP1066; 10µM) treatment.

Supplementary Figure 7

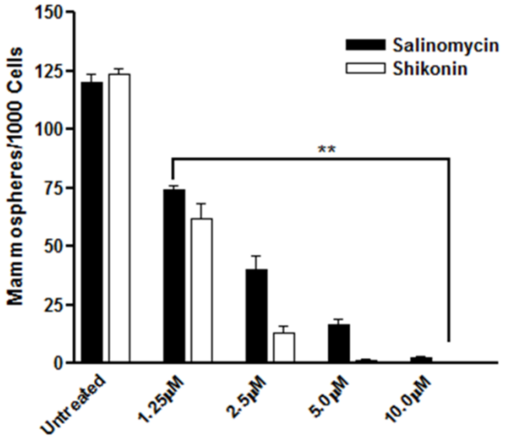

Supplementary Figure 7. Comparison of mammosphere inhibitory potential of Shk and Salinomycin. MDA-MB 231 cells were subjected to mammosphere formation in presence of indicated doses of Shk and Salinomycin for 24 h. After 24 h media was changed and cultures were grown for 8 days. The bar graph represents the average values from three independent experiments performed in duplicate. (**) $p < 0.01$.

Supplementary Figure 8

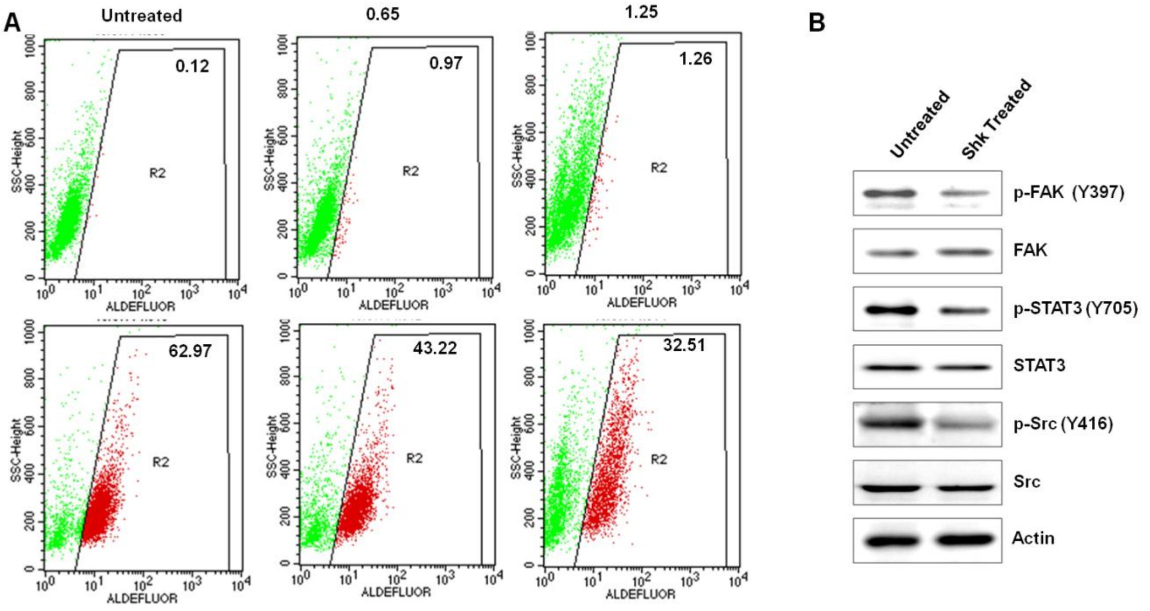

Supplementary Figure 8. Effect of Shk on 4T1 cells in-vitro. (A) 4T1 cells were treated with Shk for 24 h and ALDH1 positive cells were detected using Aldefluor assay. **(B)** Expression and activation of FAK, Src and STAT3 were measured using western blotting in control (DMSO treated) and Shk (1.25 µM) treated 4T1 cells for 3 h.

Supplementary Figure 9

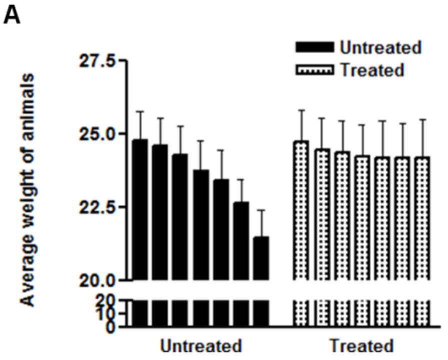

B

Groups	AST (U/L)	ALT (U/L)	BUN (mg/dL)	Hgb (mg/dL)	WBC (K/µl)	PLT (K/µl)
Vehicle	116±9.1	36±2.1	56±2.1	12.4±0.2	2.6±0.1	849±21
Shikonin	117±7.2	35±1.4	55±1.5	12.6±0.2	2.5±0.1	847±19

Supplementary Figure 9. Changes in animal weight, lung metastasis and Biochemical Blood Parameters of the mice used for 4T1-Syngenic tumor experiments on shikonin administration. (A) Average weights of mice bearing 4T1 tumors and treated with vehicle or 2.5mg Kg^{-1}shikonin. **(B)** For the analysis of biochemical blood parameters, heparinized blood samples (50 µl /mouse) were collected on day 0 and at the end of treatment by submandibular bleeding. The clinical chemistry of the blood including parameters for AST: aspartate aminotransferase; ALT: alanine aminotransferase; BUN: blood urea nitrogen; WBC: White blood cells; Hgb: hemoglobin and PLT:

Platelets were obtained using the Hemagen Analyst VR Benchtop Chemistry System (Hemagen Diagnostics, Inc. Columbia,USA). Mean ±SE of six mice per treatment group at the end of the study.

Supplementary Figure 10

Full Blots for Figure 2.

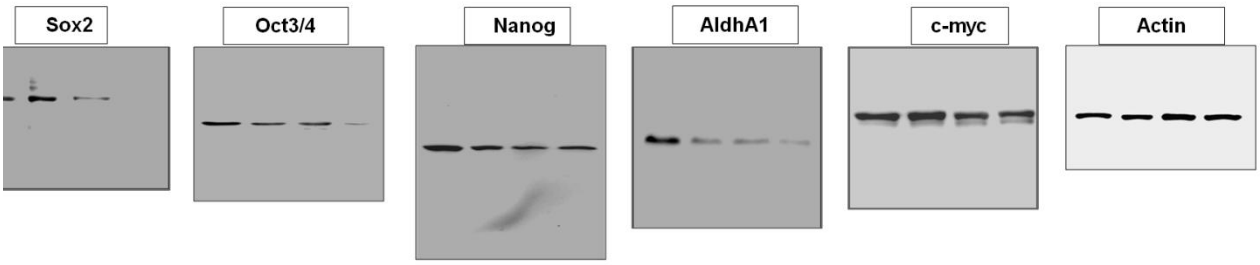

Full Blots for Figure 3.

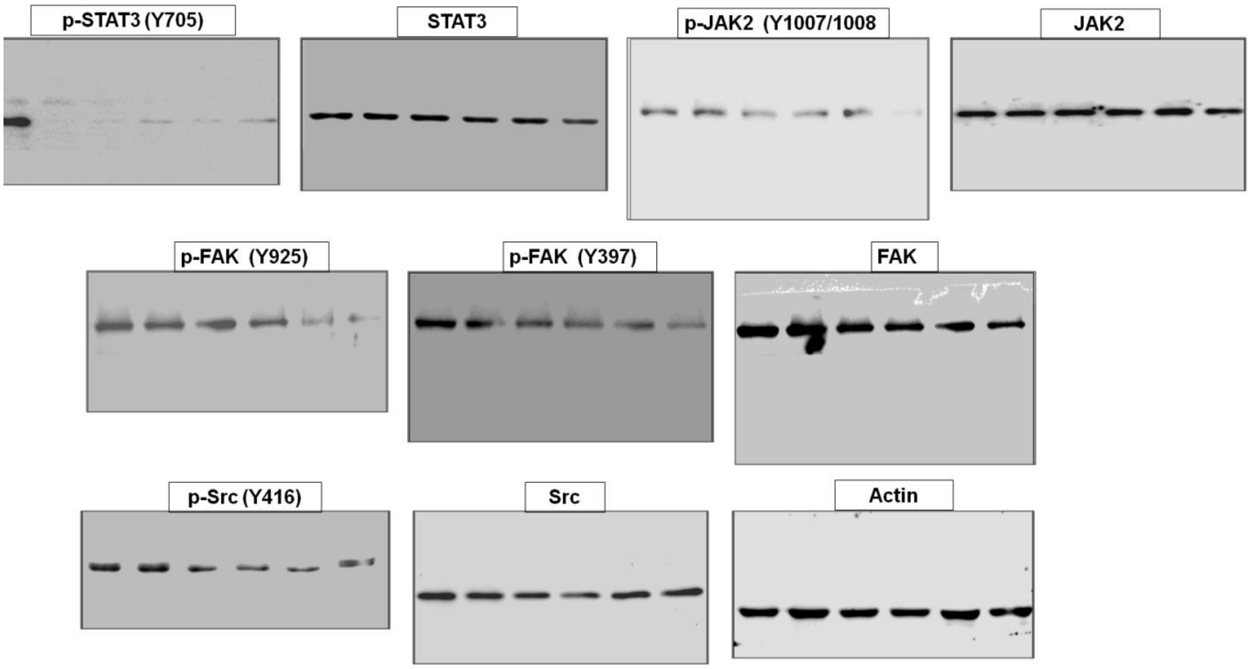

Full Blots and gels for Figure 4.

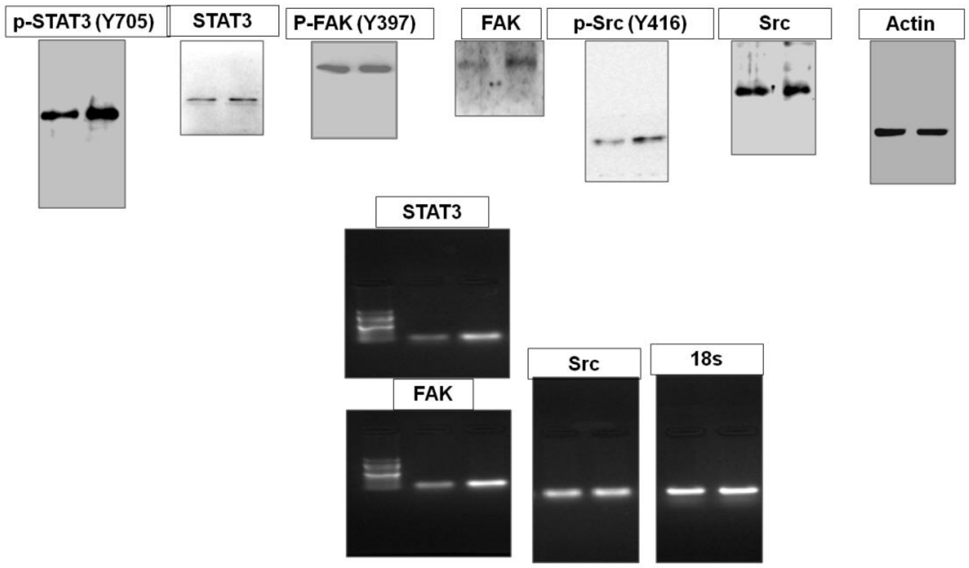

Supplementary Figure 10 Cont.

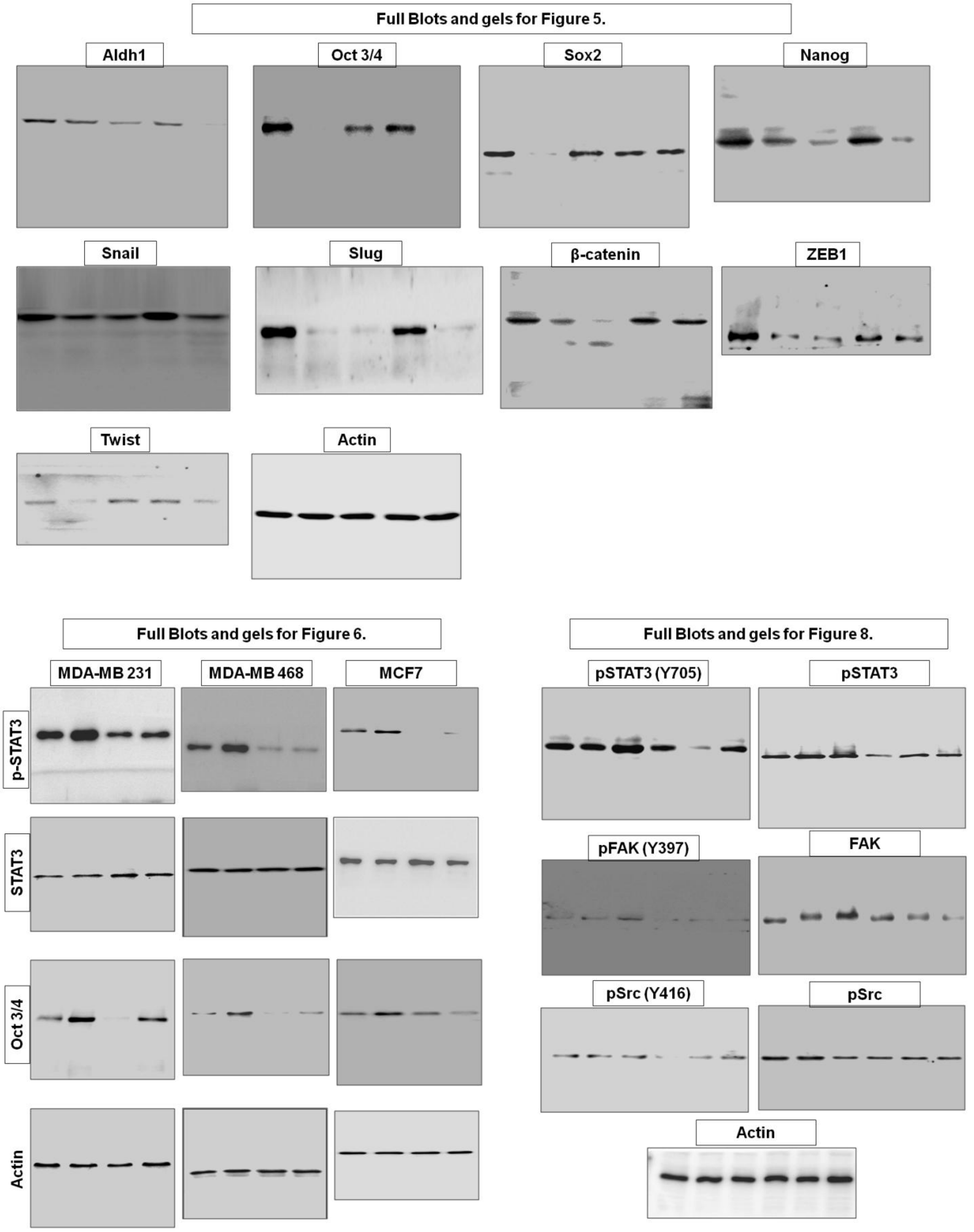

Supplementary Figure 10. Full blot and gel images.

Part 2: Supplementary Materials and Methods

PBMC Isolation: For Peripheral blood mononuclear cells (PBMCs) isolation whole blood (10 ml), anticoagulated with EDTA, was loaded carefully onto Ficoll Histopaque (Sigma) and centrifuged for 30 min at 1500 RPM (Centrifuge 5804 R; Eppendorf) without brakes at room temperature. The PBMC-containing band was aspirated and the cells were washed three times in PBS, pH 7.4, at 4°C.

MTT Assay: 3000 cells were seeded per well in 96 well plates (Nunc) and incubated at 37^{0}C for 24h. Afterwards, culture media was replaced and cells were treated with different concentration of Shk and respective amount of DMSO as indicated. Cell viability was determined by MTT assay. Absorbance was taken at 540nm (FLUOstar Omega,BMG-Lifetech) and percent cell viability was calculated.

ATPlite 1step assay: The primary breast cancer cells were seeded in white 96-well plates (Costar, MA, USA) in 100 μ L of media at varying densities to achieve 60% to 70% confluency after five days in culture. Shk was dissolved in DMSO were diluted in media containing 2% FBS prior to the treatments. In all the experiments a vehicle control corresponding to the highest DMSO concentration (not exceeding 0.2%,volume/volume). Subsequently, the cells were cultured for 24 hours, the media were aspirated and the cells were treated with various concentrations of Shk and vehicle controls in triplicates. After four days of treatment, viability was determined utilizing the ATPlite 1step assay system (Perkin Elmer, Waltham, MA, USA) according to the manufacturer’ s instructions. Luminescence measurements were acquired utilizing a Perkin Elmer 2104 EnVision plate reader. Raw luminescence values were normalized to the DMSO vehicle control wells.

Apoptosis assay using flow-cytometry: Apoptosis and cell cycle analysis was performed according to methods described elsewhere.[58] Briefly, cells were seeded in 6 well plates at the intensity of $1x10^5$ cells / well and treated with different doses of Shk as indicated for 24 hr. There after cells were trypsinized, washed with phosphate buffered saline (PBS) and resupended in 1x binding buffer

containing Annexin-V-FITC and PI for 15 minutes at room temperature. Stained cells were analyzed for live, necrotic and apoptotic cells by flow cytometer (FACS Calibur, BD Biosciences).

Primary Culture of breast cancer cells: Each sample was minced and digested at 37^0C, for 2 to 4 h in a solution of type IV collagenase, 1 mg mL^{-1} (Sigma), containing 40 mg mL^{-1} of bovine serum albumin, 2 mg mL^{-1} of glucose, 100 units mL^{-1} of penicillin and 100 mg mL^{-1} of streptomycin (P+S; Sigma), 50 mg L^{-1} of gentamicin (Sigma), and 1.25 mg/L of Fungizone (Life Technologies). After incubation, the samples were extensively rinsed with PBS and suspended in culture media. Cells were subsequently subjected to centrifugation at 40X g for generation of epithelial breast – enriched fraction. The isolated epithelial cells were seeded in 24-well plates at a density of 10^6 cells/well and cultured in MEGM epithelial cell culture media (Lonza).

Mammosphere Subculture: For subculture, mammospheres were washed with PBS and treated with Stempro Accutase to dissociate the mammosphere. Cells were centrifuged and passed through 40-µm cell strainer (BD) to get a single cell suspension. Single cells were subjected to mammosphere culture as indicated above.

CD44, CD24 Immunophenotyping: A single cell suspension containing 1×10^5 cells were resuspended in 8 µL of phycoerythrin (PE)-conjugated mouse antihuman CD24 monoclonal antibody and allophycocyanin (APC)-conjugated mouse anti-human CD24 monoclonal antibody (R&D biosystems), and incubated at 4°C in the dark for 30 to 40 min. The labeled cells were washed and then analyzed on a FACS Calibur (BD).

STAT3 Reporter Assay: The effect of Shk on the transcriptional activity of STAT-3 was studied using a CIGNAL Reporter Assay kit (SABiosciences, Frederick, MD) based dual-luciferase assays consisting of a STAT-3-specific firefly reporter and a *Renilla* luciferase normalization reporter. The assay was performed in a 24-well plate, as per the manufacturer's instructions. The Luminescence was measured in ultra-multifunctional microplate reader.

The primers sequences used in PCR and real-time qPCR:

Species	Gene Name	Primer Orientation	Primer Sequence (5'-3')
Mouse	GAPDH	Forward primer	TGCCCAGAACATCATCCCTG
Mouse	GAPDH	Reverse primer	TGAAGTCGCAGGAGACAACC
Mouse	Aldh1a1	Forward primer	CTCCTGGCGTGGTAAACATT
Mouse	Aldh1a1	Reverse primer	CCATGGTGTGCAAACTCAAC
Mouse	Oct3/4	Forward primer	AGCCGACAACAATGAGAACC
Mouse	Oct3/4	Reverse primer	TGATTGGCGATGTGAGTGAT
Mouse	Sox2	Forward primer	AAGGGTTCTTGCTGGGTTTT
Mouse	Sox2	Reverse primer	AGACCACGAAAACGGTCTTG
Mouse	Myc	Forward primer	GCCCAGTGAGGATATCTGGA
Mouse	Myc	Reverse primer	ATCGCAGATGAAGCTCTGGT
Mouse	Twist	Forward primer	CCCACACCTCTGCATTCTGA
Mouse	Twist	Reverse primer	TTTGCAGGCCAGTTTGATCC
Mouse	Nanog	Forward primer	CGCCATCACACTGACATGAG
Mouse	Nanog	Reverse primer	AGAAGAATCAGGGCTGCCTT
Human	ALDH1A1	Forward primer	CTGTGTTCCAGGAGCCGAAT
Human	ALDH1A1	Reverse primer	AGCATCCATAGTACGCCACG
Human	OCT3/4	Forward primer	GAGCAAAACCCGGAGGAGT
Human	OCT3/4	Reverse primer	TTCTCTTTCGGGCCTGCAC
Human	SOX2	Forward primer	ACACCAATCCCATCCACACT
Human	SOX2	Reverse primer	GCAAACTTCCTGCAAAGCTC
Human	C-MYC	Forward primer	CGTCCTCGGATTCTCTGCTC
Human	C-MYC	Reverse primer	GCTGCGTAGTTGTGCTGATG
Human	NANOG	Forward primer	GCTTGCCTTGCTTTGAAGCA
Human	NANOG	Reverse primer	TTCTTGACCGGGACCTTGTC

Human	ß-Catenin	Forward primer	ATGACGAGGACCAGGTGGTGGTT
Human	ß-Catenin	Reverse primer	GCAAGGTCCCAGCGGTACAACG
Human	Snail	Forward primer	TGCGCTACTGCTGCGCGAAT
Human	Snail	Reverse primer	AGGGCTGCTGGAAGGTAAACTCTGG
Human	ZEB1	Forward primer	AGGATGACACAGGAAAGGAAGGGCA
Human	ZEB1	Reverse primer	TGACCACTGGCTTCTGGTGTGC
Human	Slug	Forward primer	AGAACTCACACGGGGGAGAAGCCT
Human	Slug	Reverse primer	CACTCAGTGTGCTACACAGCAGCC
Human	TWIST1	Forward primer	GGGCCGGAGACCTAGATGTCATTGT
Human	TWIST1	Reverse primer	CGCCCCACGCCCTGTTTCTT
Human	STAT3	Forward primer	AAAGCAGCAAAGAAGGAGGC
Human	STAT3	Reverse primer	CTGGCCGACAATACTTTCCG
Human	FAK	Forward primer	AATCGGCCCAGAAGAAGGAA
Human	FAK	Reverse primer	CGCAATGGTTAGGGATGGTG
Human	Src	Forward primer	ACCATCCTCACACTGGTCAG
Human	Src	Reverse primer	AGCGTCCTCATCTGGTTTCA
Human	18s	Forward primer	GGAGAGGGAGCCTGAGAAAC
Human	18s	Reverse primer	CCTCCAATGGATCCTCGTTA
